# Forced Degradation Studies of Ivabradine and *In Silico* Toxicology Predictions for Its New Designated Impurities

**DOI:** 10.3389/fphar.2016.00117

**Published:** 2016-05-04

**Authors:** Piotr Pikul, Marzena Jamrógiewicz, Joanna Nowakowska, Weronika Hewelt-Belka, Krzesimir Ciura

**Affiliations:** ^1^Department of Physical Chemistry, Faculty of Pharmacy with the Subfaculty of Laboratory Medicine, Medical University of GdańskGdańsk, Poland; ^2^Department of Analytical Chemistry, Chemical Faculty, Gdańsk University of TechnologyGdañsk, Poland; ^3^Mass Spectrometry and Chromatography Laboratory, Pomeranian Science and Technology ParkGdynia, Poland

**Keywords:** ivabradine, stress testing, stability, LC-MS/MS, *in silico*, ADME/Tox calculations

## Abstract

All activities should aim to eliminate genotoxic impurities and/or protect the API against degradation. There is a necessity to monitor impurities from all classification groups, hence ivabradine forced degradation studies were performed. Ivabradine was proved to be quite durable active substance, but still new and with insufficient stability data. Increased temperature, acid, base, oxidation reagents and light were found to cause its degradation. Degradation products were determined with the usage of HPLC equipped with Q-TOF-MS detector. Calculations of pharmacological and toxicological properties were performed for six identified degradation products. Target prediction algorithm was applied on the basis of Hyperpolarization-activated cyclic nucleotide-gated cation channels, as well as more general parameters like logP and aqueous solubility. Ames test and five cytochromes activities were calculated for toxicity assessment for selected degradation products. Pharmacological activity of photodegradation product (**UV4**), which is known as active metabolite, was qualified and identified. Two other degradation compounds (**Ox1** and **N1**), which were formed during degradation process, were found to be pharmacologically active.

## Introduction

Guidelines for assessing the quality of active pharmaceutical ingredient (API) and medicinal products has been developed by ICH, FDA, WHO or EMA, which are focused to the greatest extent, firstly to verify the stability of the API through the establishment of various tests and the way there are conducted (ICH Q1A, 1993). Secondly, the confirmation of the presence of impurities is performed (ICH Q3A, 1993). Studies are performed to explore various paths of the potential degradation of the API, evaluating the rate of changes under the influence of various factors.

For a comprehensive evaluation process associated with the pharmaceutical stability, there is a need to perform various tests, which results should be the answer whether the drug substance is stable. The most important for the drug substance are so-called stress tests, which involve overly intense exposure to factors such as: strong acid, base, hydrogen peroxide, high temperature or light, in a manner individual to the different drugs (ICH Q1A (R2), 2003; Waterman and Adami, 2005). When the chemical stability of APIs is considered, the effects of chemical reactions of the API are evaluated, mainly: hydrolysis in conditions of increased humidity, oxidation in the presence of oxygen or hydrogen peroxide, isomerization, hydration, dimerization, or decarboxylation. Photostability tests are characterized by particular specificity and are an integral part of stability tests that are included in standard, ICH Q1A (1993). It is worth noting that the concept of photodegradation is related not only to changes in the structure of the API under the influence of light, but also to the occurrence of free-radical processes, energy transfer, or even luminescence, which may lead to unexpected and atypical results, especially in the solid state (Glass et al., 2004). There is a special need to recognize all possibilities of degradation of new pharmaceutical compound especially those, which are not recognized in Pharmacopeia yet.

Determination of unknown impurities in the new drug compound is based on the results obtained during various stress tests, thus during chemical stability evaluation. Identification of degradation products is primarily concentrated on establishing its structure, with simultaneous determination of physicochemical properties, and then toxicity estimation accordingly to the latest recommendations (Carstensen and Rhodes, 2000; EMEA, 2004; Olsen and Larew, 2005; Huynh-Ba, 2008). The first ICH regulations regarding the quantitation limit of impurities in drug, were based solely on a patient daily dose of API, routes of administration and duration of therapy and the identification of impurity of concentration below 0.1% was unrequired (ICH Q3B (R), 2000). In addition, there was also the lack of awareness of impurities existing in the drug substance itself, but in the finished product mainly. In 2004 there has been a noticeable change in the approach to standards and thresholds of impurities, as a reaction to the proposal developed by the EMEA, in which necessity for determination of the genotoxic impurities limits in the API was stated (EMEA, 2004; Modi et al., 2012). It has been proposed that the lowest possible quantitation limit of impurities should be enforced in cases of the predicted presence of a genotoxic substance formed from an API (Dow et al., 2013; Maggio et al., 2013). The latest guidelines has been developed in 2014 and requirement to use at least two forecasting models of mutagenicity predicting *in silico* was established.

According to current recommendations, it is important to provide a lot of results in the area of stability testing research in case of newly implemented pharmaceutical compounds as well as others already known (Jamrógiewicz, 2016). Ivabradine (**IVA**) is a new drug compound in the treatment of heart failure patients and the only implemented HCN4 channel inhibitor. The drug received an expedited review under the FDA’s priority review program and the approval was based on results of the shift trial, published in 2010. Locking HCN4 channel results in slower growth of pacemaker current and as a result reducing the heart rate. Such action is used to treat angina, especially in patients with intolerance to β-blockers. **IVA** is metabolized by cytochrome CYP3A4 and does not affect metabolism and plasma concentration of other cytochome inhibitors. Ivabradine is a highly soluble *S*-enantiomer with no *in vivo* conversion. The only known active metabolite is *N*-desmethyl ivabradine (DiFrancesco, 2010; EMA, 2015). There are no officially published impurities for **IVA** in pharamacopeias. The only results focused on degradation and *in silico* studies have been performed recently (Patel et al., 2015). So that, it is important to provide an independent point of view and new data.

There are many models and approaches of *in silico* studies (Singh et al., 2012, 2013). In this work, Prediction SwissTarget and ePhysChem tools were applied (Gfeller et al., 2014; ePhysChem, 2015). By using the above-mentioned software, we evaluated the toxicity of some identified degradation products. They were defined by physicochemical parameters such as logP and solubility, which determine the pharmacokinetics. The **IVA** impact on cytochrome P450 (a group of enzymes with oxidase activity responsible among other things for the detoxification of the body) has also been examined (Danielson, 2002). The major enzyme from the group of cytochrome P450 isoenzymes is CYP3A4, responsible for the metabolism of most drugs or xenobiotics. Drugs can be metabolized by different enzymes such as CYP2D6, CYP2C9, CYP2C19, and CYP1A2, therefore, different metabolites are formed, which activity may be similar to the original drug substance. Sometimes these products cause toxic effects (Zanger and Schwab, 2013; Patel et al., 2015).

Physicochemical parameters such as logP and solubility of degradation products are discussed in this work because of their importance in perspective of pharmacokinetics (Alavijeh et al., 2005; Bergström, 2005). If the drug is administered orally, as ivabradine, it is exposed to low pH in the stomach, which may form chlorides drug substance with altered properties (Bergström, 2005).

The main object of this work is to conduct a forced degradation of ivabradine and propose its possible degradation products. Stress tests were carried out mainly in context of resistance to the oxidizing agent, light as well as acid and base hydrolysis. Additionally, the calculated physicochemical, pharmacological and toxic properties of possible degradation products were presented.

## Materials and Methods

### Chemicals

The tested ivabradine hydrochloride (purity ≥ 99%) bulk powder was supplied by Watson International Ltd. (Kunshan, China). Acetonitrile was purchased from Sigma-Aldrich Chemical CO. (St. Louis, MO, USA), ammonium acetate, hydrochloric acid and sodium hydroxide all pure p. a. were purchased from POCH (Gliwice, Poland). 30% Hydrogen peroxide was supplied by J. T. Baker, (Deventer, The Netherlands). Pure water was obtained from Direct-Q3 UV-R Ultrapure water purification system, Merck Millipore (Darmstadt, Germany).

### Equipment and Conditions

LC-MS/MS analysis was performed with HPLC Agilent 1100 (AgilentTechnologies, Santa Clara, CA, USA) equipment coupled with QSTAR XL (AbSciex, Framingham, MA, USA) mass spectrometer. Electrospray ionization (ESI) was operated in a positive ion mode in order to obtain soft and efficient ionization of ivabradine and its degradation products. Kromasil 100 C8 (4.6 mm × 250 mm, 5 μm, AkzoNobel, Amsterdam, Netherlands) was used in reversed-phase mode with isocratic elution. Mobile phase composition was 65% of component A (20 mM ammonium acetate) and 35% of component B (acetonitrile). Total analysis time was 30 min. Column temperature throughout the analysis was 25°C the flow rate of the mobile phase was 1 ml/min and the injection volume was 20 μl. The high resolution Q-TOF mass spectrometer was operated in SCAN mode to obtain mass spectrum in the mass range 220–1000 m/z. MS/MS mass spectra were obtained by collision-induced dissociation of selected parent ions in Product Ion mode of mass spectrometer. Degradation studies were carried out in Thermostat CC2-K6 made by Huber company (Offenburg, Germany), photo stability studies were performed in a photostability Suntest + Atlas chamber (Accelerated Tabletop Exposure Systems) with xenon lamp. Shimadzu UV-1800 UV-Vis spectrophotometer was also used in the studies.

### Stress Testing

In order to prepare degradation sample, for each of the samples 1mg of ivabradine were weighed and dissolved in 2 ml of the appropriate solvent for particular test sample.

Thermal degradation was performed by adding deionized water and kept for 24 h in 80°C. To achieve an acid and alkaline hydrolysis a 2 M HCl solution and 1 M NaOH were added, respectively, respectively and incubated for 24 h in 80°C. Studies of possible oxidation products were carried out by adding 3% H_2_O_2_, 7.5% H_2_O_2_ and 15% H_2_O_2_ and incubated for 24 h in 80°C. Photolytic degradation was performed in solution of deionized water for 24 and 48 h as well as in solid form for 120 h. The illuminance was set at 500 W/m^2^.

### *In Silico* Calculations

Ivabradine is metabolized by cytochrome P450. To calculate the probable metabolites, Toxtree program was used (Toxtree, 2015), applying SMARTCyp method to predict the fragments in the molecule, which are the most susceptible to activity of cytochrome P450.

For the calculation of toxicity (CYP3A4, CYP2D6, CYP2C9, CYP2C19, and CYP1A2), logP, solubility and Ames test, ePhysChem (2015) was used, while the prediction of degradation compounds on potassium/sodium hyper polarization activated cyclic nucleotide-gated channel 4 (HCN4) were counted using SwissTargetPrediction (Gfeller et al., 2014). Calculations were made for **IVA** and compounds **N1**, **Ox1**, **Ox4**, **Ox5**, **UV1,** and **UV2**.

## Results and Discussion

### Metabolism/Degradation Products Prediction

**FIGURE 1:**
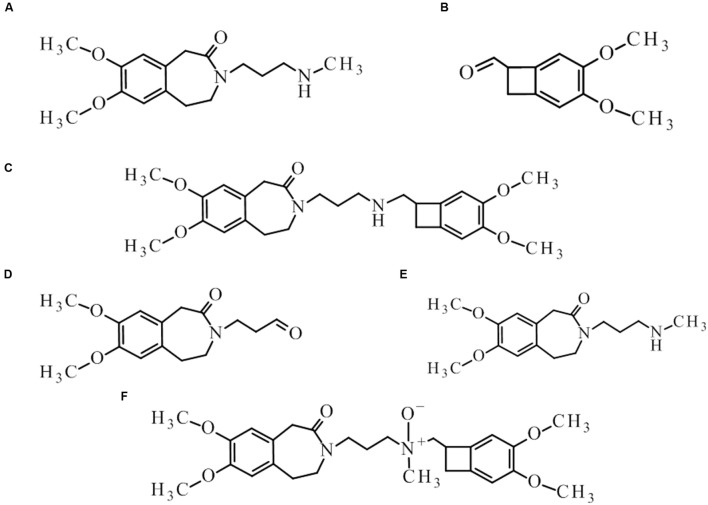
**Theoretical metabolites: main (rank 1) – **(A–C)** and secondary (rank 3 and > 4) – (**D–F**) generated by the usage of software Toxtree**.

Cytochrome P450 is an enzyme, which demonstrates a monooxygenase activity (Zanger and Schwab, 2013). In the absence of these enzymes, but with an appropriate amount of supplied energy as heat, redox potential, a strong electrolyte, or UV radiation, reactions can occur *in vitro* to form the same or similar degradation products. Using the structure of the molecule and the Toxtree software (Toxtree, 2015), theoretical metabolites which may be produced by the action of cytochrome P450 were calculated (**Figure 1**).

These theoretical degradation products formed by the *N*-demethylation (**Figures 1A–E**) and *N*-oxidation (**Figure 1F**) are presented in **Figure 1**.

### Chromatogram Evaluation

**FIGURE 2:**
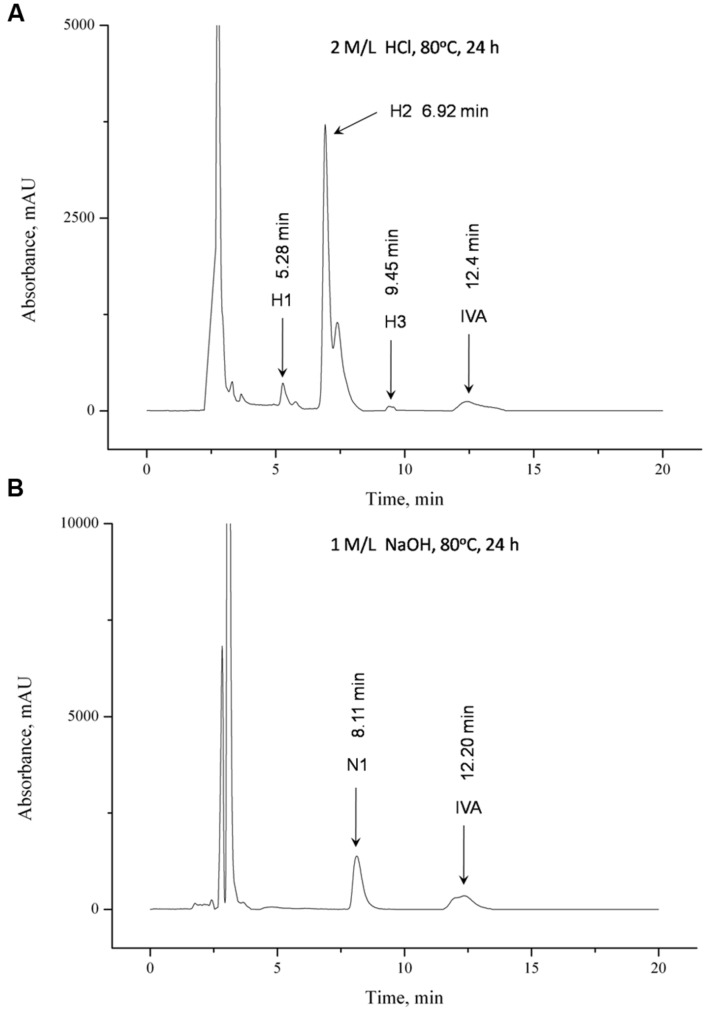
**Chromatograms obtained by the HPLC separation of products of **(A)** acid hydrolysis and **(B)** alkaline hydrolysis of ivabradine**.

Ivabradine was tested with the addition of HCl and NaOH (**Figures 2A,B**), wherein the concentration of used HCl was greater than the concentration of used NaOH. On the chromatogram obtained from a sample after acidic hydrolysis reaction of **IVA** (**Figure 2A**) a satisfactory separation of ivabradine and the degradation products is observed. Only compound **H2** was not separated from other neighboring unidentified peak. Separation of the basic hydrolysis degradation products of ivabradine was achieved with satisfactory result (**Figure 2B**).

**FIGURE 3:**
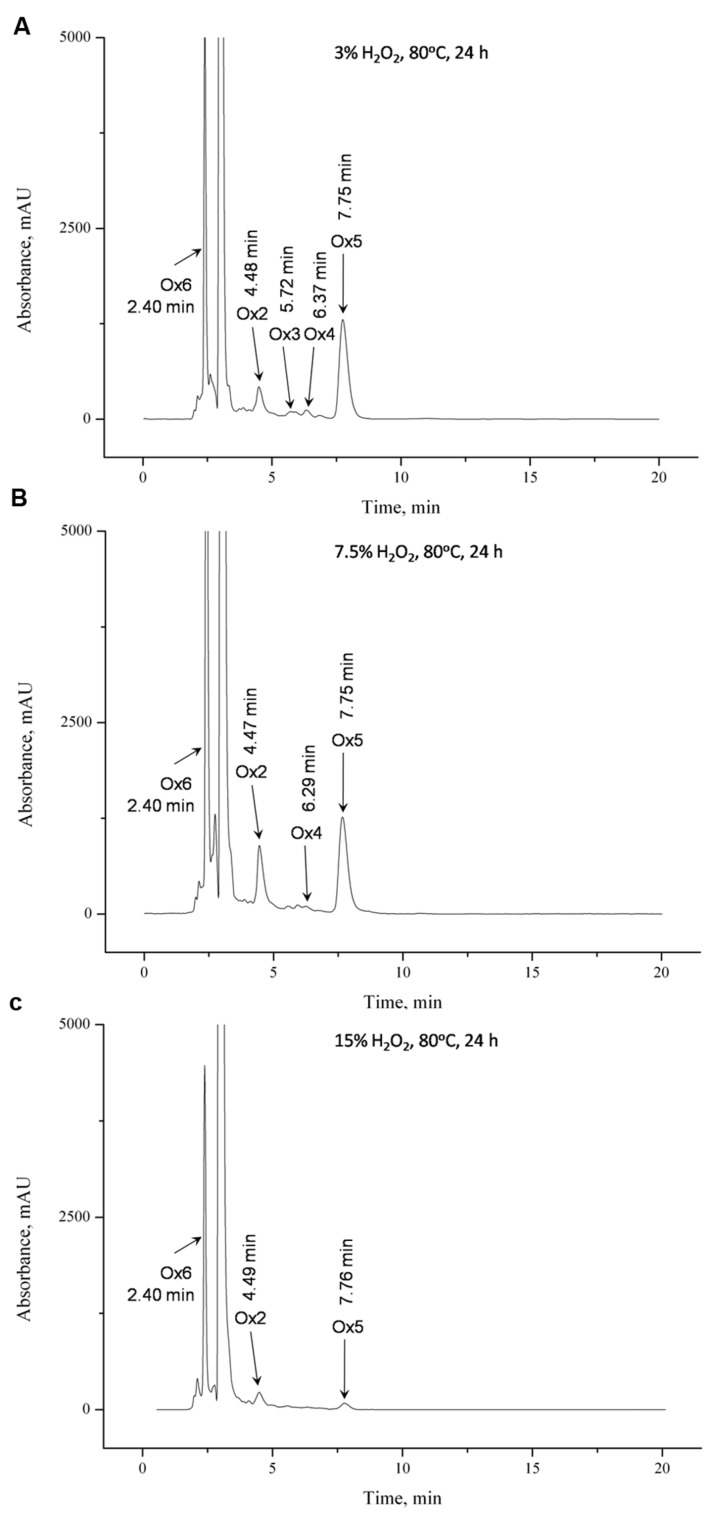
**HPLC chromatogram showing effect on the addition of different amounts of H_2_O_2_**(A)** 3%, **(B)** 7.5%, and **(C)** 15%**.

Complete degradation of **IVA** was observed in all samples containing hydrogen peroxide (**Figures 3A–C**). Chromatographic separation of degradation products was achieved. It was noted, that peaks corresponding to the products **Ox5** and **Ox4** are smaller in case the higher concentration of hydrogen peroxide is used in respective samples.

**FIGURE 4:**
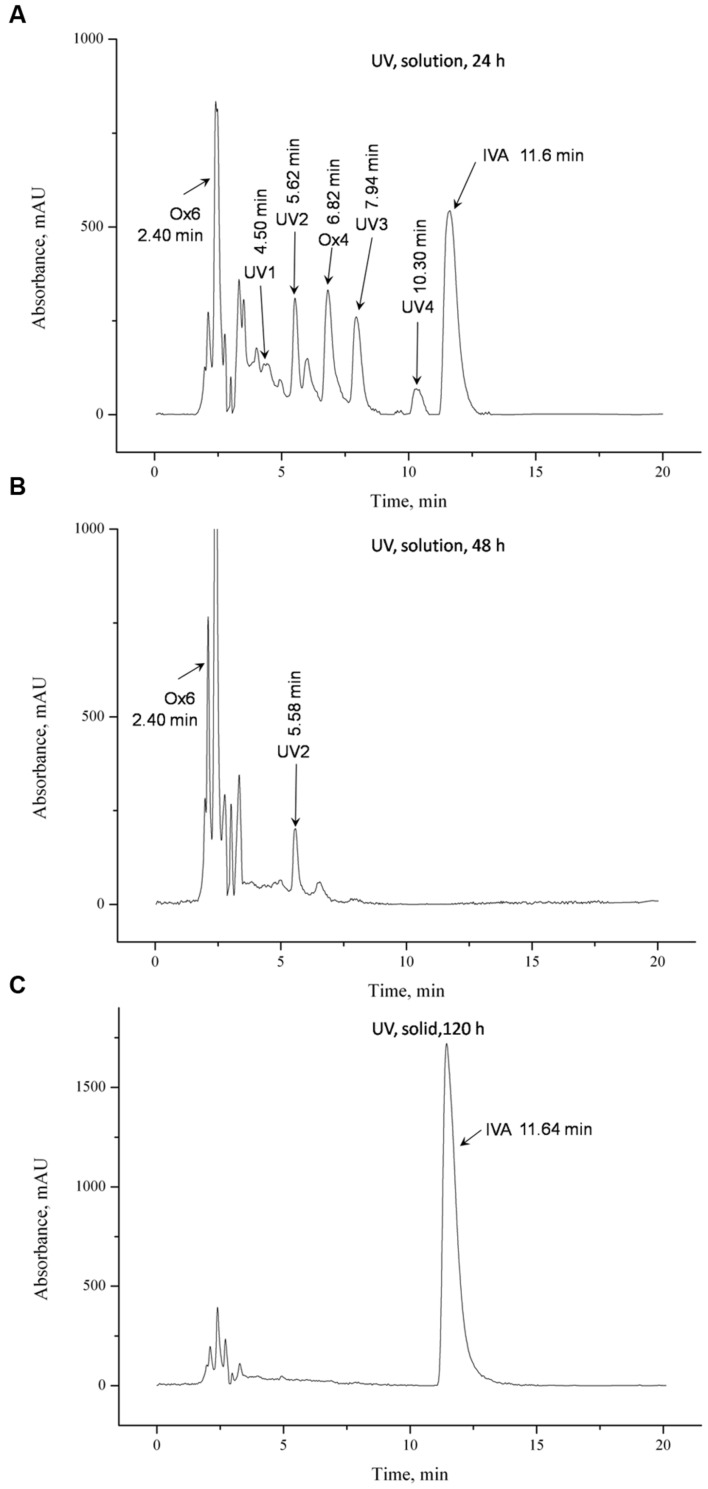
**HPLC chromatogram showing photolytic degradation in solution (**A**) for 24 h, **(B)** 48 h, and **(C)** in solid for 120 h**.

During 24 h photodegradation of **IVA**, six degradation products were produced (**Figure 4A**). 48-h exposure to UV radiation caused complete decomposition of **IVA** in solution (**Figure 4B**) as well as reduction of the intense of peak corresponding to the product **UV2**. Peaks separation in each UV-tested samples were satisfactory. A lot of photoproducts in samples and relatively short time of analysis resulted in insufficient separation of compound **UV1**. Solid powder form of ivabradine, exposed to irradiation for 120 h, was proved to be durable, because any degradation product on the chromatogram was reveled (**Figure 4C**).

Determination of degradation products is presented in **Table 1**. Differences in retention times and distorted shape of the peaks corresponding to ivabradine compound tested in hydrolytic as well as photolytic reaction, is probably caused by the formation of enantiomers during degradation processes.

**Table 1:** Degradation products of all tested samples.

Sample	Degradation products	Ivabradine [min]
Acid decomposition	H1, H2, H3	12.40
Alkaline decomposition	N1	12.20
Oxidation (3% H_2_O_2_)	Ox1, Ox2, Ox3, Ox4, Ox5	–
Oxidation (7.5% H_2_O_2_)	Ox1, Ox2, Ox4, Ox5	–
Oxidation (15% H_2_O_2_)	Ox1, Ox2, Ox5	–
Photolysis (24 h)	Ox1, UV, UV2, Ox4, UV3, UV4,	11.60
Photolysis (48 h)	Ox1, UV2	–
Photolysis (powder 120 h)	–	11.64


### Degradation Product Identification

Using the above described apparatus and conditions, MS/MS analyses were performed for all chromatographic peaks (Francois-Bouchard et al., 2000; Lu et al., 2012).

**FIGURE 5:**
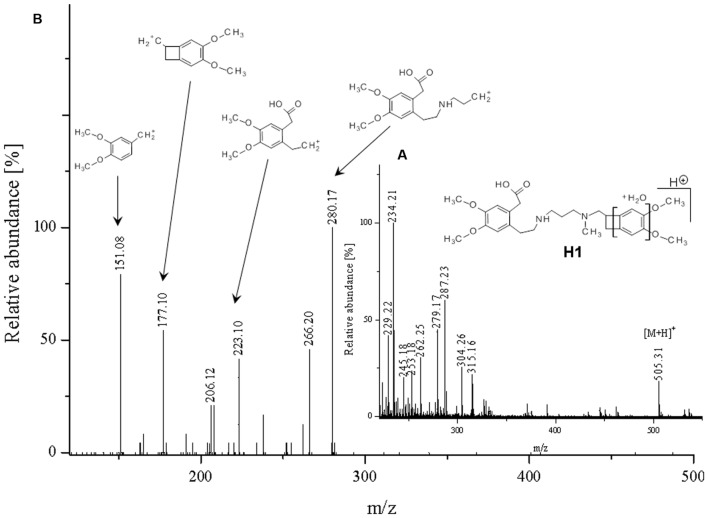
**Proposed structure and MS/MS fragmentation pattern of H1.** **(A)** MS spectrum and **(B)** MS/MS spectrum of [M+H]^+^ ion at 505.31 *m/z*.

**FIGURE 6:**
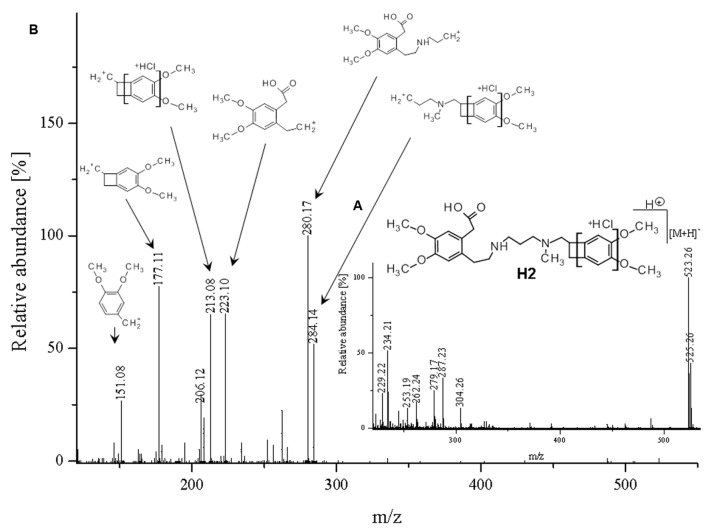
**Proposed structure and MS/MS fragmentation pattern of H2.** **(A)** MS spectrum and **(B)** MS/MS spectrum of [M+H]^+^ ion at 523.26 *m/z*.

**FIGURE 7:**
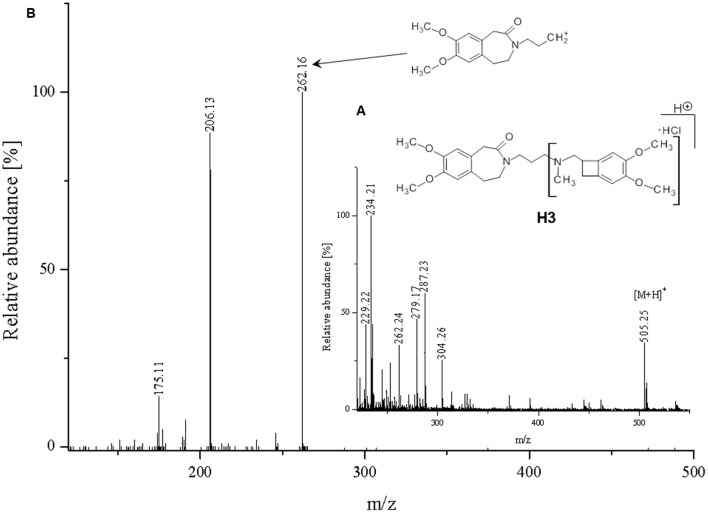
**Proposed structure and MS/MS fragmentation pattern of H3.** **(A)** MS spectrum and **(B)** MS/MS spectrum of [M+H]^+^ ion at 505.25 *m/z*.

**FIGURE 8:**
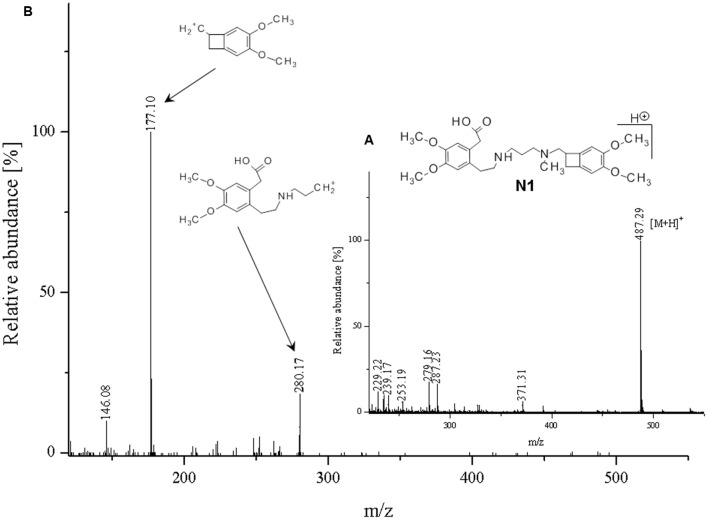
**Proposed structure and MS/MS fragmentation pattern of N1.** **(A)** MS spectrum and **(B)** MS/MS spectrum of [M+H]^+^ ion at 487.29 *m/z*.

**FIGURE 9:**
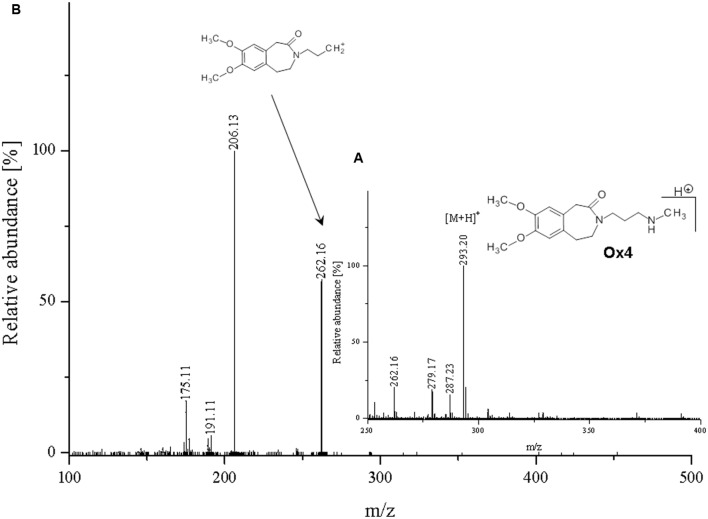
**Proposed structure and MS/MS fragmentation pattern of Ox4.** **(A)** MS spectrum and **(B)** MS/MS spectrum of [M+H]^+^ ion at 293.20 *m/z*.

**FIGURE 10:**
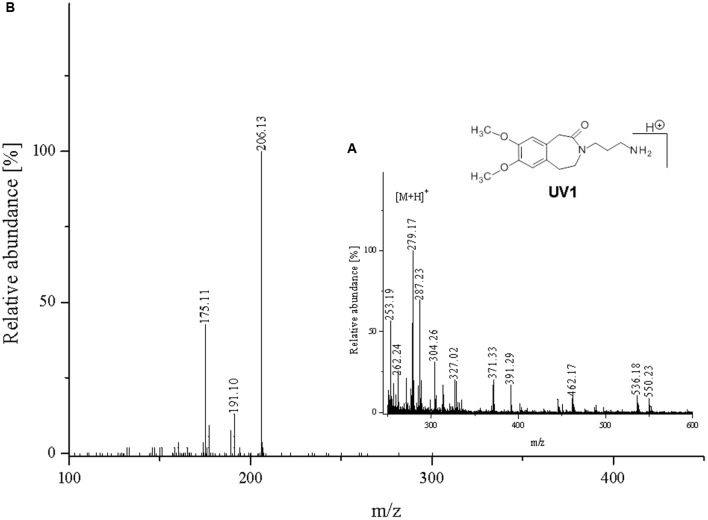
**Proposed structure and MS/MS fragmentation pattern of UV1.** **(A)** MS spectrum and **(B)** MS/MS spectrum of [M+H]^+^ ion at 279.17 *m/z*.

**FIGURE 11:**
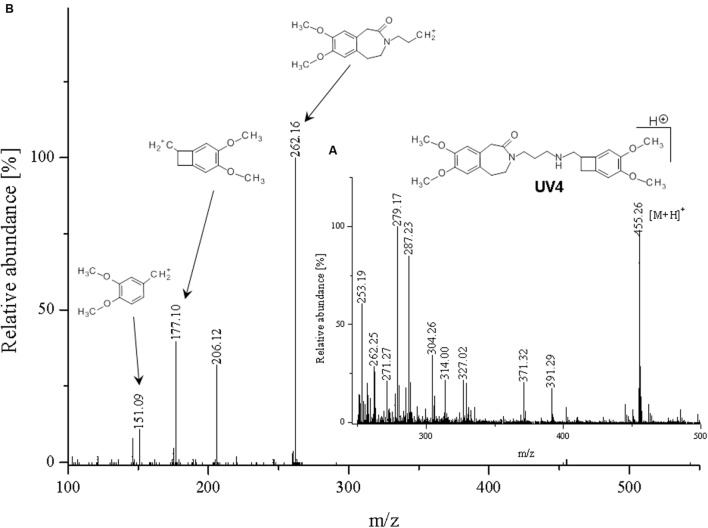
**Proposed structure and MS/MS fragmentation pattern of UV4.** **(A)** MS spectrum and **(B)** MS/MS spectrum of [M+H]^+^ ion at 455.26 *m/z*.

The resulting MS/MS spectra with the proposed fragmentation patterns are presented in **Figures 5–11**. The proposed structures of degradation products together with other experimental data are presented in **Table 2**. The accuracy of the experimental mass degradation products with the theoretical masses are shown in **Table 3**.

**Table 2:** Comparison of structural formulas, chemical formulas, retention times, molecular ions, as well as the fragment ions of the products obtained in the experiment.

No	Structural formula	Chemical formula	Retention time [min]	[M+H]^+^	Fragment ions^a^
**Hydrolysis**
H1	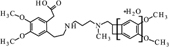	C_27_H_40_ClN_2_O_7_	HCl – 5.28	505	151, 177, 206, 223, 266, 280
H2	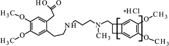	C_27_H_39_ClN_2_O_6_	HCl – 6.92	523	177, 206, 213, 223, 280, 284
H3	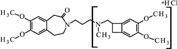	C_27_H_37_ClN_2_O_5_	HCl – 9.45	505	206, 262
N1	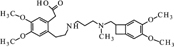	C_27_H_38_N_2_O_6_	NaOH – 8.11	487	177, 280
**Oxidation**
Ox1	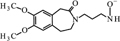	C_15_H_21_N_2_O_4_	3% H_2_O_2_ – 2.40 7.5% H_2_O_2_ – 2.40 15% H_2_O_2_ – 2.40 UV 24 h – 2.40 UV 48 h – 2.40	294	175, 191, 206
Ox2	Unknown	3% H_2_O_2_ – 4.48 7.5% H_2_O_2_ – 4.47 15% H_2_O_2_ – 4.49	Inconclusive
Ox3	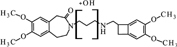	C_27_H_34_N_2_O_6_	3% H_2_O_2_ – 5.72	471	177, 192, 206, 248
Ox4	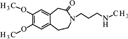	C_16_H_24_N_2_O_3_	3% H_2_O_2_ – 6.37 7.5% H_2_O_2_ – 6.29 UV 24 h – 6.82	293	175, 206, 262
Ox5	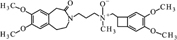	C_27_H_36_N_2_O_6_	3% H_2_O_2_ – 7.75 7.5% H_2_O_2_ – 7.68 15% H_2_O_2_ – 7.76	485	177, 206, 262
**Photolysis**
xUV1	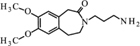	C_15_H_22_N_2_O_3_	UV 24 h – 4.5	279	175, 191, 206
UV2	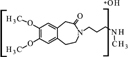	C_16_H_24_N_2_O_4_	UV 24 h – 5.56 UV 48 h – 5.58	309	204, 260, 278
UV3	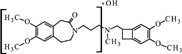	C_27_H_36_N_2_O_6_	UV 24 h – 7.94	485	146, 177, 204, 278
UV4	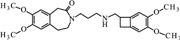	C_26_H_34_N_2_O_5_	UV 24 h – 11.6	455	151, 177, 206, 262


**Table 3:** Measured and calculated mass of molecular ion and fragmentation ion of degradation products, with calculated mass error.

Monoisotopic mass	Measured	Theoretical	Mass error in m/z
IVA [M+H]^+^	469.2775	469.2697	-0.0078
	262.1509	262.1438	-0.0071
	177.1053	177.0910	-0.0143
H1 [M+H]^+^	505.2908	505.3073	0.0165
	280.1543	280.1684	0.0141
	223.0965	223.1020	0.0055
	177.0901	177.1003	0.0102
	151.0754	151.0814	0.0060
H2 [M+H]^+^	523.2590	523.2569	0.0021
	284.1424	284.1412	0.0012
	280.1684	280.1543	0.0141
	223.1020	223.0965	0.0055
	213.0758	213.0677	0.0081
	177.1053	177.0901	0.0152
	151.0814	151.0754	0.0060
H3 [M+H]^+^	505.2482	505.2464	0.0018
	262.1631	262.1438	0.0193
N1 [M+H]^+^	487.2875	487.2803	-0.0072
	280.1684	280.1543	-0.0141
	177.1003	177.0901	-0.0102
Ox1 [M+H]^+^	294.1384	294.1580	0.0196
Ox3 [M+H]^+^	471.2718	471.2490	-0.0228
	248.1322	248.1281	-0.0041
	177.0951	177.0910	-0.0041
Ox 4 [M+H]^+^	293.2008	293.1860	-0.0148
	262.1570	262.1438	-0.0132
Ox5 [M+H]^+^	485.2667	485.2646	-0.0021
	262.1502	262.1438	-0.0064
	177.0901	177.0910	0.0009
UV1 [M+H]^+^	279.1682	279.1703	0.0021
UV2 [M+H]^+^	309.1932	309.1809	-0.0123
	278.1543	278.1387	-0.0156
UV3 [M+H]^+^	485.2814	485.2646	-0.0168
	278.1543	278.1387	-0.0156
	177.1003	177.0901	-0.0102
UV4 [M+H]^+^	455.2592	455.2540	-0.0052
	262.1570	262.1438	-0.0132
	177.1003	177.0901	-0.0102
	151.0860	151.0754	-0.0106


The ketone group on the benzoazepine ring in compound **H1** has been oxidized to a carboxyl group (**Figure 5**). The characteristic peak m/z = 280 on the mass spectrum corresponding to the open azepine ring near the nitrogen, is observed for three of the four products of hydrolysis (**Figures 5**, **6**, and **8**). Water and HCl near the benzocyclobutene in compounds **H1** and **H2** are attached without a covalent bond, a kind of adducts may be formed. The situation is similar for the product **H3**, except that here the ring benzoazepine is intact (no peak of m/z = 280) and the fragmentation spectrum of MS/MS proved there is impossible sufficiently formation of the HCl adduct closely to the molecule (**Figure 7**). Compound **N1**, as another product of ivabradine hydrolysis (**Figure 8**) differs only by oxidized carboxyl group on the benzoazepine ring near the nitrogen, what is observed as opening of the ring.

Due to the high level of noise and discrepancies in the resulting MS/MS spectrum of compound **Ox2**, its identification is impossible, but due to repetition of its occurrence, there is importance to place attention on that compound in stability testing of ivabradine.

Limitations of the MS-Q-TOF method used for identification of degradation products of **IVA** did not allow for a clear determination of the attachment site of the hydroxyl group in compound **Ox3**, but it is possible the covalent bond forms between nitrogen atoms in the molecule.

Compounds **Ox4** and **Ox1** have similar masses (m/z 293.20 and 294.1388, respectively) as well as the fragmentation spectra. Differences are in the presence of diverse substituents near the nitrogen atom in the molecule chain, a methyl group in **Ox4** (**Figure 9**), and oxygen in **Ox1**. Oxygen is coupled to the nitrogen atom also in case of compound **Ox5**, where it forms *N*-oxide bond.

Compound **UV1** (**Figure 10**) is the degradation product of the smallest identified molecular weight identified of **IVA** (m/z = 279.1725). For compounds **UV2** and **UV3** it is impossible to predict its structures, but the data suggest that OH group is joined in the region of benzoazepine ring. Compound UV4 is varies from ivabradine by the lack of the methyl group on the nitrogen atom (**Figure 11**). It is proved, that compound **UV4** is a metabolite of ivabradine produced by cytochrome P450.

### ADME/Tox Screening for Degradation Products

The calculation of pharmacological and toxicological properties could be done only for six ivabradine degradation products, since its chemical structures managed to establish for obtained data. In other degradation products we proposed the most probable chemical structures only on the basis of MS/MS analysis.

The pharmacological effect of ivabradine is associated with blocking of HCN4 channels (Postea and Biel, 2011). Therefore, in order to make comparative analysis calculations were also done for ivabradine, even though the drug is introduced into clinical practice. The *in silico* calculations made for ivabradine and selected degradation products demonstrated high probability of HCN4 channel inhibition for **Ox4**, **Ox5**, **UV1**, and **UV4**, which may indicate a certain pharmacological action of these products (**Table 4**). For a compound **UV4** calculated value of inhibition is very high (0.94) and only slightly lower than calculated value for the ivabradine (0.96). It is known that compound **UV4** is a metabolite of ivabradine with proved ability to inhibit HCN4 channels. Other studies have shown that it is much weaker inhibitor than ivabradine (DiFrancesco, 2010).

**Table 4:** ADME/Tox calculations for ivabradine and selected degradation products.

			[19]	[18]	[18]	[18]	[18]

**No.**	**[M+H]^+^**	**SMILES**	**HCN4 inhibition^a^**	**CYP2D6 inhibition**	**CYP3A4 inhibition**	**logP_o/w_**	**Aqueous solubility**
IVA	468	CN(CCCN1CCc2cc (c(cc2CC1 = O)OC)OC) CC3Cc4c3cc(c (c4)OC)OC	0.96^b^	Inhibitor (57.0%^c^)	Inhibitor (74.0%^c^)	3.18 Log unit ± 0.38^d^	3.51 -log(mol/L) ± 0.70^d^
N1	487	CN(CCCNCCc1cc (c(cc1CC( = O)O) OC)OC)CC2Cc3c2cc (c(c3)OC)OC	0.28^b^	Non-inhibitor (65.0%^c^)	Non-inhibitor (84.0%^c^)	2.71 Log unit ± 0.38^d^	2.39 -log(mol/L) ± 0.70^d^
Ox1	294	COc1cc2c(cc1OC) CC( = O)N(CC2) CCCN[O-]	0.40^b^	Non-inhibitor (84.0%^c^)	Non-inhibitor (86.0%^c^)	0.57 Log unit ± 0.74^d^	0.98 -log(mol/L) ± 0.70^d^
Ox4	293	CNCCCN1CCc 2cc(c(cc2CC1 = O)OC)OC	0.82^b^	Non-inhibitor (69.0%^c^)	Non-inhibitor (89.0%^c^)	1.23 Log unit ± 0.38^d^	1.36 -log(mol/L) ± 0.70^d^
Ox5	485	C[N+](CCCN1CC c2cc(c(cc2CC1 = O) OC)OC)(CC3Cc4c3cc (c(c4)OC)OC)[O-]	0.77^b^	Non-inhibitor (79.0%^c^)	Inhibitor (62.0%^c^)	1.26 Log unit ± 0.74^d^	2.39 -log(mol/L) ± 0.70^d^
UV1	279	COc1cc2c (cc1OC)CC( = O) N(CC2)CCCN	0.79^b^	Non-inhibitor (85.0%^c^)	Non-inhibitor (87.0%^c^)	0.42 Log unit ± 0.38^d^	0.9 -log(mol/L) ± 0.70^d^
UV4	455	COc1cc2c(cc1OC) CC( = O)N(CC2) CCCNCC3Cc4c3cc (c(c4)OC)OC	0.94^b^	Non-inhibitor (59.0%^c^)	Inhibitor (76.0%^c^)	2.43 Log unit ± 0.38^d^	3.03 -log(mol/L) ± 0.70^d^


*^a^Potassium/sodium hyperpolarization-activated cyclic nucleotide-gated channel 4; ^b^probability; ^c^accuracy; ^d^within a confidence interval of 66%.*

Ivabradine and its degradation products showed no mutagenicity in the Ames test. It was also shown, that no inhibition of cytochromes: CYP2C9, CYP2C19, and CYP1A2 (data calculated but not included in **Table 4**) occurs for all products of degradation and ivabradine itself. According to obtained calculations only ivabradine inhibits CYP2D6. Some authors refer only to the inhibition of CYP3A4 by **IVA** (DiFrancesco, 2010; Postea and Biel, 2011), which is confirmed by our calculations. Compound **UV4,** which has lower pharmacological activity than ivabradine, demonstrated inhibition of cytochrome, the same as compound **Ox5**.

The values of log P and aqueous solubility, allow to divide identified degradation products into three groups. Degradation products of ivabradine - compounds **UV4** and **N1** have slightly reduced values of log P and solubility in comparison to **IVA**. In the case of degradation products **Ox4** and **Ox5,** significant reduction of lipophilicity is observed. A strong decrease in lipophilicity and aqueous solubility occurred in the case of compounds **Ox1** and **UV1**. Significant reduction of the log P values can be explained by an aromatic ring being highly lipophilic moiety.

## Conclusion

Stress testing of ivabradine was presented with successful identification and characterization of its degradation products. The obtained compounds showed no mutagenic effects in performed *in silico* models, in which toxicity and the effect on cytochromes seems to be small. Another important aspect was, that their lower water solubility and lipophilicity in comparison to ivabradine, may influence their bioavailability after oral administration. Above all, lower pharmacological activity of degradation products was proven in calculated value of channel inhibition HCN4. There was also computationally confirmed pharmacological activity of compound **UV4**.

## Author Contributions

PP: co-operation in the development of research area and methodology; co-operation in the collecting the literature; coordination in the manuscript preparation; co-operation in MS/MS spectra analysis; Toxtree analysis; MJ: co-operation in the manuscript preparation; co-operation in MS/MS spectra analysis; co-operation in the collecting the literature; corresponding author; JN: co-operation in the development of research area and methodology; WH-B: LC-MS/MS method development; LC-MS/MS spectra recording; KC: Toxtree analysis; co-operation in the manuscript preparation.

## Conflict of Interest Statement

The authors declare that the research was conducted in the absence of any commercial or financial relationships that could be construed as a potential conflict of interest.
